# Nuclear Targeting of IGF-1 Receptor in Orbital Fibroblasts from Graves' Disease: Apparent Role of ADAM17

**DOI:** 10.1371/journal.pone.0034173

**Published:** 2012-04-10

**Authors:** Neil Hoa, Shanli Tsui, Nikoo F. Afifiyan, Amiya Sinha Hikim, Bin Li, Raymond S. Douglas, Terry J. Smith

**Affiliations:** 1 Divisions of Molecular Medicine and Endocrinology, Department of Medicine, Harbor-University of California Los Angeles Medical Center, Torrance, California, United States of America; 2 David Geffen School of Medicine at University of California Los Angeles, Los Angeles, California, United States of America; 3 Veterans Affairs Medical Center, Long Beach, California, United States of America; 4 Departments of Ophthalmology and Visual Sciences and Internal Medicine, University of Michigan Medical School, Ann Arbor, Michigan, United States of America; Cardiff University, United Kingdom

## Abstract

Insulin-like growth factor-1 receptor (IGF-1R) comprises two subunits, including a ligand binding domain on extra- cellular IGF-1Rα and a tyrosine phosphorylation site located on IGF-1Rβ. IGF-1R is over-expressed by orbital fibroblasts in the autoimmune syndrome, Graves' disease (GD). When activated by IGF-1 or GD-derived IgG (GD-IgG), these fibroblasts produce RANTES and IL-16, while those from healthy donors do not. We now report that IGF-1 and GD-IgG provoke IGF-1R accumulation in the cell nucleus of GD fibroblasts where it co-localizes with chromatin. Nuclear IGF-1R is detected with anti-IGF-1Rα-specific mAb and migrates to approximately 110 kDa, consistent with its identity as an IGF-1R fragment. Nuclear IGF-1R migrating as a 200 kDa protein and consistent with an intact receptor was undetectable when probed with either anti-IGF-1Rα or anti-IGF-1Rβ mAbs. Nuclear redistribution of IGF-1R is absent in control orbital fibroblasts. In GD fibroblasts, it can be abolished by an IGF-1R-blocking mAb, 1H7 and by physiological concentrations of glucocorticoids. When cell-surface IGF-1R is cross-linked with ^125^I IGF-1, ^125^I-IGF-1/IGF-1R complexes accumulate in the nuclei of GD fibroblasts. This requires active ADAM17, a membrane associated metalloproteinase, and the phosphorylation of IGF-1R. In contrast, virally encoded IGF-1Rα/GFP fusion protein localizes equivalently in nuclei in both control and GD fibroblasts. This result suggests that generation of IGF-1R fragments may limit the accumulation of nuclear IGF-1R. We thus identify a heretofore-unrecognized behavior of IGF-1R that appears limited to GD-derived fibroblasts. Nuclear IGF-1R may play a role in disease pathogenesis.

## Introduction

The insulin-like growth factor-1 receptor (IGF-1R) is a membrane spanning protein through which IGF-1 and IGF-2 exert many of their actions [1]. It comprises two subunits [1], [2]. The ligand binding, extracellular domain of IGF-1R is located on IGF-1Rα while the membrane-spanning β subunit contains the tyrosine phosphorylation site necessary for canonical signal initiation. IGF-1R functions to support cell growth [3] and transformation [4]. Its activation can lead to the generation of anti-apoptotic signals including several cell-survival proteins [5], [6]. Besides its importance in the regulation of metabolism, IGF-1R can determine the quality and magnitude of immune responses and may play a role in the pathogenesis of autoimmunity [7]. Most studies examining IGF-1R function have focused on the activation of orthodox kinase signaling pathways [1], [8]. In addition to their activities on the cell membrane surface, several other transmembrane tyrosine kinase receptors have been found to translocate to the cell nucleus and in so doing influence gene expression [9], [10]. But intracellular trafficking of the IGF-1R to the cell nucleus has not been reported previously.

Graves' disease (GD) is an autoimmune process where the thyroid gland becomes enlarged and over-active [11]. Activating IgGs directed against the thyrotropin receptor (TSHR), termed thyroid-stimulating antibodies (TSI) or GD-derived IgG (GD-IgG), drive thyroid gland over-activity and accelerated tissue metabolism through cyclic AMP generation [12]. In addition, connective tissues in the orbit become activated, inflamed, and undergo substantial remodeling in a process known as thyroid-associated ophthalmopathy (TAO) [13], [14]. Reports have appeared suggesting that some relationship exists between levels of TSI and the clinical activity of TAO [15]. Although conceptually appealing, participation of these antibodies in the pathogenesis of TAO has yet to be firmly established [16].

A key aspect of GD concerns the recruitment of T lymphocytes and other pro-inflammatory cells to involved anatomic sites [17], [18]. We recently reported that fibroblasts from patients with GD become activated by their GD-IgG and synthesize two powerful T lymphocyte chemoattractants, IL-16 and RANTES [19]. This response is mediated through over-expressed IGF-1R [20]. In contrast, control fibroblasts from donors without known autoimmune disease fail to exhibit this response. We postulate that GD-IgG activation of orbital fibroblasts in GD results in tissue infiltration with T and B lymphocytes [18], [21], [22], cells that also over-express IGF-1R in GD [23], [24]. TSHR and IGF-1R form a physical and functional complex in GD fibroblasts and thyroid epithelial cells [25]. This may account for at least some of the tissue responses to TSH. The activation of Erk by TSH can be attenuated with IGF-1R-blocking antibodies [25]. Thus it is possible that GD-IgG targeting of not only TSHR but also IGF-1R plays a pathological role in GD. Besides IL-16 and RANTES, GD fibroblasts from the orbit treated with IGF-1 and GD-IgG also generate higher levels of hyaluronan than untreated controls [26].

Here we report that the higher level of cell surface-displayed IGF-1R on TAO fibroblasts is associated with the accumulation of an IGF-1R fragment in the cell nucleus. This trafficking of endogenous IGF-1R does not occur in fibroblasts from healthy donors. It is completely blocked by 1H7, an anti-IGF-1R blocking antibody, and is dependent on the activity of ADAM17, a metalloproteinase involved in the shedding of surface proteins [27], [28]. It appears that nuclear trafficking of IGF-1R represents a previously unrecognized phenotypic signature peculiar to orbital fibroblasts from patients with TAO that may therefore underlie GD-specific responses to IGF-1 and GD-IgG.

## Results

### IGF-1 and GD-IgG provoke nuclear accumulation of IGF-1Rα in GD-Fibroblasts

Cultured human fibroblasts express IGF-1R [29]. Orbital fibroblasts from patients with TAO are skewed toward the IGF-1R^+^ phenotype and levels of the receptor protein are increased compared to those from healthy controls [20], [25]. This increased receptor density exhibits durability over many population doublings and serial passages *in vitro*. When GD orbital fibroblasts or those from control donors are treated with IGF-1 (10 nM), loss of cell-surface IGF-1R can be detected by flow cytometric analysis. The magnitude of this reduction is appreciable: IGF-1 lowered the fraction of cells expressing the receptor by 46%. It could be blocked by lowering the temperature of the cells from 25°C to 4°C and by treating cultures with dexamethasone (10 nM) (data not shown).

**Figure 1:**
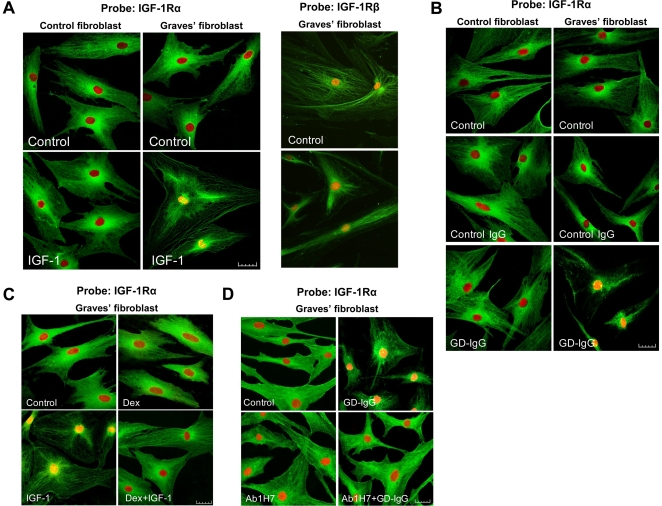
Confocal imaging of IGF-1R reveals nuclear accumulation in TAO fibroblasts following treatment with either IGF-1 or GD-IgG. (**A**) untreated (control, upper panels) or IGF-1 (10 nM) (lower panels) for 16 h. reveals localization of IGF-1Rα (green) as indicated by the yellow overlay nuclei in GD fibroblasts. Chromatin was stained by PI (red). Control fibroblasts failed to respond. The images labeled IGF-1Rβ (green) demonstrate an absence of effects with IGF-1 on nuclear accumulation. Scale bar, 25 µm. (**B**) IGF-1Rα (green) co-localizes in the PI-stained nuclei (yellow overlay) following treatment with GD-IgG (15 µg/ml) in GD but not in control fibroblasts. (**C**) Dexamethasone (10 nM) attenuates the nuclear accumulation of IGF-1Rα (**D**) as does the IGF-1R-blocking mAb, 1H7 (5 µg/ml). These experiments have been performed 4 times.

**Figure 2:**
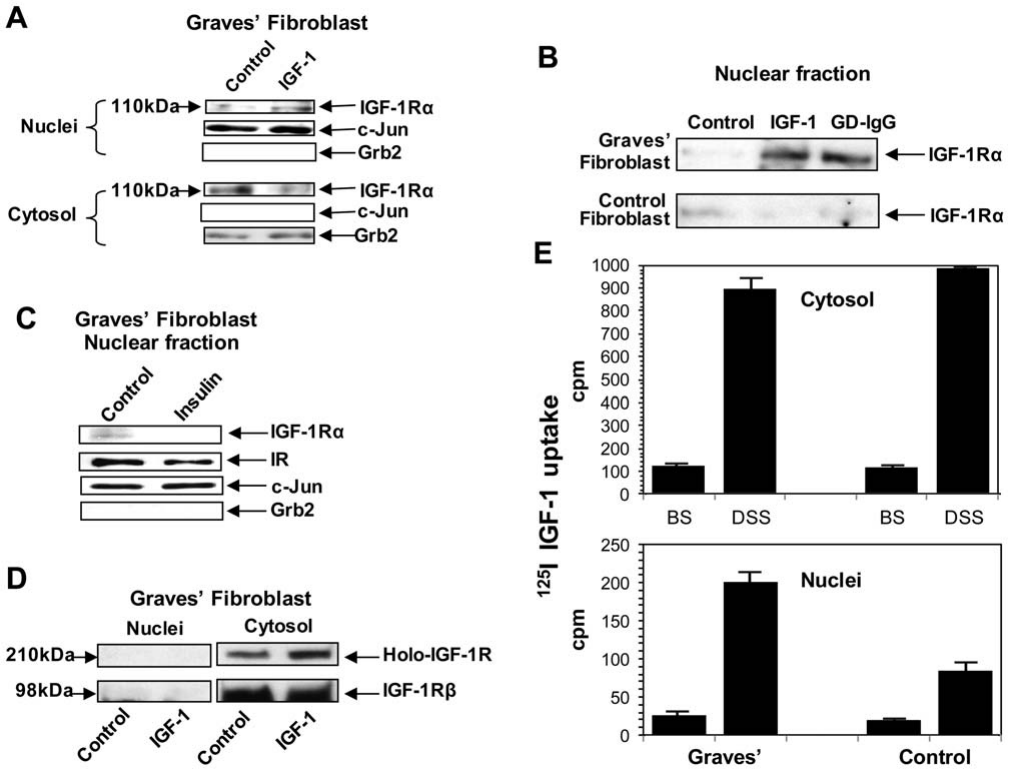
IGF-1R protein differentially accumulates in the nuclei of TAO orbital fibroblasts and derives from the fibroblast surface. (**A**) Western blot analysis of nuclear and cytoplasmic IGF-1Rα in GD orbital fibroblasts before and following IGF-1 (10 nM) treatment for 16 h. Cells were subjected to subcellular fractionation as described in “Methods” and membranes were probed with anti-IGF-1Rα, stripped, and re-probed with anti-Grb2 (cytoplasmic) and anti-c-Jun (nuclear) Abs. (**B**) Nuclear IGF-1Rα content in GD and control orbital fibroblasts before or following treatment with either IGF-1 (10 nM) or GD-IgG (15 µg/ml) for 16 hours. (**C**) Insulin fails to alter the nuclear content of IR or IGF-1Rα in GD orbital fibroblasts. Cells were treated with nothing or insulin (15 µg/ml) for 16 hrs. They were subjected to subcellular fractionation and Western blot analysis. (**D**) IGF-1Rβ (98 kDa) and the intact receptor (200 kDa) are undetectable in the nucleus under basal and IGF-1-treated conditions. (**E**) Control and GD fibroblasts were subjected to ^125^I-IGF-1 cross-linking with either the cell-impermeable agent, BS, or the cell permeable agent, DSS. They were then treated with IGF-1. Nuclei were separated as described in “Methods” and subjected to quantification of radioactivity. Results are representative of three experiments performed.

To assess IGF-1R trafficking from the surface of orbital fibroblasts, cells were stained and subjected to confocal immunofluorescense microscopy. Chromatin was stained with propidium iodide (PI, red). IGF-1Rα was detected using a FITC conjugated secondary antibody against rabbit polyclonal anti-IGF-1Rα antibody (green) (Fig. 1A). IGF-1R staining localized on the plasma membrane and in the central cytoplasm of untreated control and GD fibroblasts. In striking contrast to the pattern in untreated cells, those incubated with IGF-1 accumulated IGF-1R in the cell nucleus (appearing yellow) while the intensity of the cytoplasmic signal was reduced (Fig. 1A). Eighty percent of nuclei in IGF-1-treated GD fibroblasts exhibited change to yellow color. The receptor remained localized primarily in the cytosol of IGF-1-treated control fibroblasts while only 5% were yellow in color. IGF-1R also appears to translocate from cytosol to the nucleus in response to GD-IgG (15 µg/ml) of GD fibroblasts, a response that was absent in control fibroblasts (Fig. 1B). Thus, the effects of IGF-1 resemble those of GD-IgG. Control IgG had no effects on IGF-1R localization (Fig. 2B). Dexamethasone (10 nM) could block the effect of IGF-1 on nuclear accumulation of IGF-1Rα (Fig. 1C). This steroid concentration is associated with near-maximal responses in human fibroblasts [30]. The IGF-1R blocking monoclonal antibody, 1H7 (5 µg/ml), which precludes receptor activation [31], was added to GD fibroblasts cultures without or with GD-IgG. As Fig. 1D demonstrates, 1H7 completely attenuated the nuclear accumulation of IGF-1Rα. Thus, it would appear that the effects of GD-IgG on IGF-1R localization are mediated directly through IGF-1R itself. This is confirmed by the ability of Des _1–3_ IGF-1 to mimic the effects of IGF-1 and GD-IgG (data not shown). That growth factor analogue acts very specifically on the receptor and has relatively low affinity for IGF-1 binding proteins [32]. The images in the right sided panels of Fig. 1A demonstrate the impact of IGF-1 on the distribution of IGF-1Rβ (green). Unlike IGF-1Rα, IGF-1Rβ fails to accumulate in the nucleus but rather is dispersed peripherally before and following IGF-1 treatment.

### IGF-1R protein accumulates in GD fibroblast nuclei following treatment with IGF-1 or GD-IgG as determined by subcellular fractionation

We next determined whether the treatment of GD fibroblasts with IGF-1 resulting in the redistribution of IGF-1R by confocal microscopy led to nuclear accumulation of receptor protein as assessed following subcellular fractionation. Fibroblasts treated with nothing (control) or IGF-1 for 16 h were then subjected to Western immunoblot analysis. The level of nuclear IGF-1R migrating as a 110 kDa band was substantially increased following treatment while a reciprocal loss was detected from the cytosol (Fig. 2A). Moreover, GD-IgG like IGF-1 increased nuclear content of IGF-1R in GD fibroblasts but had no effect in control cells (Fig. 2B). IGF-1R and the insulin receptor (IR) share both structural and functional similarities. To rule out cross-reactivity between these two proteins and to determine whether the effects of IGF-1 on IGF-1R were specific, monolayers were treated with insulin (15 µg/ml) for 16 hours. Nuclear IGF-1Rα levels were unaffected by insulin in fractionated cells (Fig. 2C). IR levels were invariant with respect to treatment with either insulin or IGF-1 (data not shown). Thus, the effects of IGF-1 on nuclear IGF-1R abundance appear to be specific.

IGF-1R detected in the nucleus could comprise intact receptor protein with an expected mw of approximately 200 kDa or a fragment containing the epitope recognized by anti-IGF-1Rα. As the Western blot analysis in Fig. 2D demonstrates, while the intact receptor was detected in the cytosolic fraction, this 200 kDa IGF-1R could not be detected by immunoblot analysis in the nucleus. This analysis utilized an anti-IGF-1Rβ monoclonal antibody and thus both 110 kDa and 200 kDa bands were absent in the nuclear compartment while both were abundant in the cytosol. Coupled with our inability to detect IGF-1Rβ associated with the nucleus using confocal microscopy, it would appear that the receptor fragment accumulating in the nucleus does not contain the epitope identified by anti-IGF-1Rβ.

**Figure 3:**
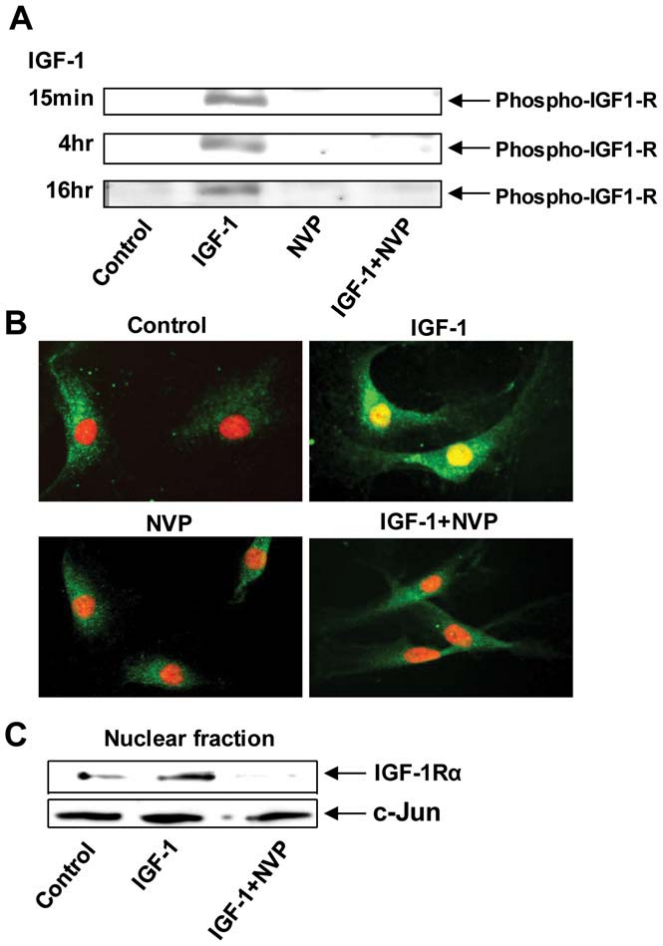
IGF-1Rβ phosphorylation may play an essential role in the nuclear translocation of IGF-1Rα in GD orbital fibroblasts. (**A**) NVP-AEW-541, a specific tyrosine kinase inhibitor blocks IGF-1R phosphorylation (**B**) NVP-AEW-541 blocks nuclear IGF-1Rα accumulation. GD orbital fibroblasts were treated as indicated and stained with anti-IGF-1Rα Ab (green) and counterstained with PI. (**C**) Western blot analysis of nuclear proteins from GD orbital fibroblasts treated with nothing, IGF-1, NVP-AEW-541, or the combination and probed with anti- IGF-1Rα and anti-c-jun Abs. The findings are representative of three experiments performed.

We next determined whether the translocation of IGF-1Rα was dependent on IGF-1R tyrosine phosphorylation. NVP-AEW541 is a novel, pyrrolo [2,3-d]pyrimidine derivative, and highly-selective inhibitor of IGF-1R kinase activity [33], [34]. When added with IGF-1, NVP-AEW541 inhibited IGF-1R phosphorylation (Fig. 3A). Moreover, the agent blocked nuclear co-localization with chromatin (Fig. 3B) and the accumulation of IGF-1R in the nucleus (Fig. 3C). This finding suggests that IGF-1R phosphorylation is essential for nuclear trafficking.

### Cell-surface [^125^I] IGF-1R complexes translocate to the nuclei of IGF-1-treated GD fibroblasts

Studies examining endogenous IGF-1R localization, as described above, relied on specific monoclonal antibodies directed against this protein. We next examined nuclear translocation with an additional approach, one that was independent of antibodies. [^125^I]-IGF-1 was cross-linked to cell surface IGF-1R displayed on GD fibroblasts. After 16 hr, fibroblasts were subjected to subcellular fractionation. As Fig. 2E demonstrates, IGF-1-bound radioactivity accumulates in the nuclei of GD fibroblasts when cross-linked to IGF-1R with a cell-permeable agent (DSS). In contrast, when an impermeable cross-linking compound (BS) was used, virtually no nuclear radioactivity could be detected. Substantially less [^125^I]-IGF-1 localized in nuclei of control fibroblasts than that detected in the GD fibroblasts. These findings suggest strongly that IGF-1Rα detected in the nuclei of IGF-1-treated GD fibroblasts originated at the cell surface.

### Activity of ADAM17 may be essential to nuclear translocation of cell-surface- associated IGF-1R

**Figure 4:**
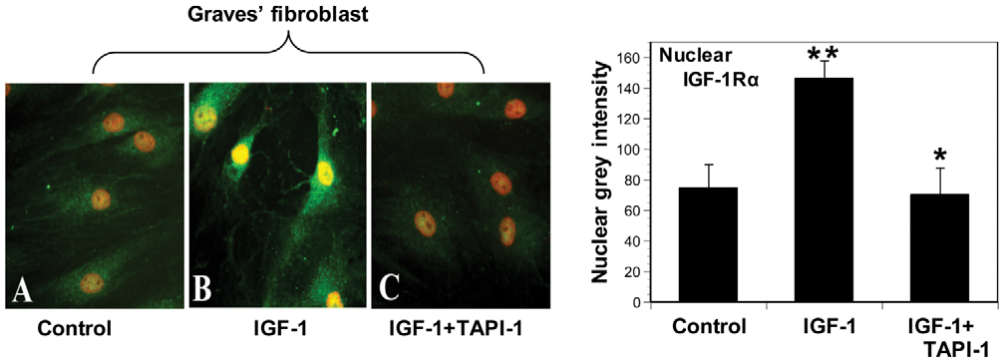
ADAM 17 appears to play a critical role in nuclear accumulation of IGF-1Rα. (**A**) GD orbital fibroblasts express ADAM 17 protein as assessed by Western blot. (**B**). TAPI-1, a specific inhibitor of ADAM17 activity, blocks the nuclear accumulation of IGF-1Rα provoked by IGF-1 in GD orbital fibroblasts. (**C**) Quantification of nuclear grey color signal intensity. Each bar in the histogram represents the mean ± SD of ten nuclei randomly chosen for each treatment group. *, p<0.05 vs IGF-1; **, p<0.01 vs control, n = 3 independent determinations.

**Figure 5:**
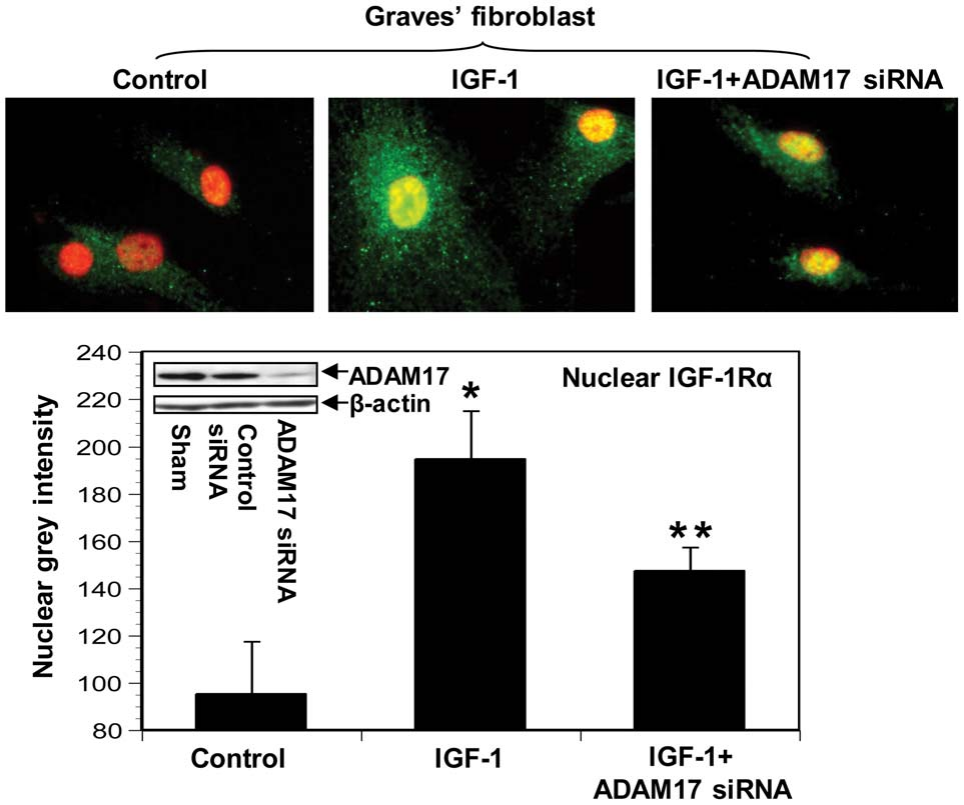
siRNA directed against ADAM17 attenuates the nuclear accumulation of IGF-1Rα in GD orbital fibroblasts. Fibroblasts were transfected with control siRNA or that specifically targeting ADAM 17. Some were left untreated while others were treated with IGF-1. Cells were fixed and stained with anti-IGF-1Rα Ab/Orgeon Green anti-rabbit Ab. Quantification of nuclear intensity was conducted so that each column represents the mean of ten randomly chosen nuclei ± SD. *, p<0.002 vs control; **, p<0.01 vs IGF-1. n = 3 independent determinations. (Inset) Adam17 siRNA or control siRNA was transfected into GD fibroblasts, cells were lysed, and proteins subjected to Western blot analysis by probing with anti-ADAM 17 Ab, stripping the membrane and incubating with anti-β-actin.

The divergence between nuclear accumulation of endogenous IGF-1Rα in control and GD fibroblasts suggests that fundamental aspects of IGF-1R protein trafficking/signaling might differ in the two cell types. Because nuclear IGF-1R following IGF-1 treatment appears to represent a fragment of the receptor lacking the epitope recognized by anti-IGF-1Rβ, we wondered whether translocation might involve the actions of cell surface proteases. One such candidate is ADAM17, a transmembrane glycoprotein metalloproteinase, involved in interactions with multiple substrates [27], [28], [35]. GD fibroblasts express high levels of ADAM17 mRNA as do control fibroblasts (control, 0.98±0.095 vs TAO, 1.0±0.08 normalized fold-change, NS). To explore its potential involvement in the fragmentation of IGF-1R, TAPI-1, a specific inhibitor of ADAM17, was added to culture medium alone or in combination with IGF-1. This agent completely blocked IGF-1R accumulation in the nucleus (Fig 4A and 4B). ADAM 17 expression was then knocked down with a specific siRNA, a maneuver that also significantly diminished nuclear IGF-1Rα accumulation (Fig. 5). In aggregate, our findings suggest that ADAM17 might play a role in the nuclear translocation of fragmented IGF-1R.

### Equivalent nuclear accumulation of virally encoded IGF-1Rα/GFP fusion protein in GD and control fibroblasts

**Figure 6:**
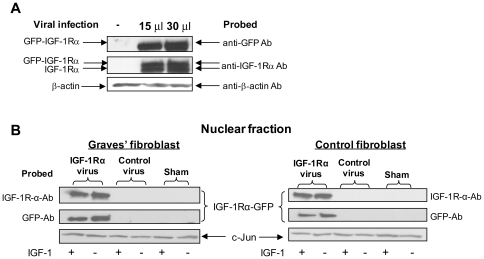
Virally encoded IGF-1Rα-GFP fusion protein expression in GD and control orbital fibroblasts. (**A**) Expression of the adenovirus-encoded IGF-1Rα–GFP fusion protein can be detected by Western blot analysis probed by both anti-GFP and anti-IGF-1Rα. (**B**) IGF-1Rα-GFP fusion protein accumulates in the nuclei of both control and GD orbital fibroblasts, as assessed by Western blot analysis following subcellular fractionation of nuclear protein. IGF-1 treatment fails to alter this pattern. These studies are representative of 5 experiments involving expression of virally encoded IGF-1Rα.

If less receptor fragmentation in control fibroblasts accounts for the absence of IGF-1Rα accumulation in the nucleus of control fibroblasts, expressing the free subunit in control and GD fibroblasts should result in equivalent nuclear IGF-1Rα. To directly test this hypothesis, a virally encoded IGF-1Rα/GFP fusion protein was cloned and transfected into fibroblasts. A dose-dependent increase in GFP- IGF-1Rα fusion protein can be detected by subjecting cell lysates to Western blot analysis (Fig. 6A). Strong co-localizing nuclear signals for both GFP and IGF-1Rα were detected in both control and GD fibroblasts (Fig. 6B). Moreover, accumulation of the fusion protein was identical in control and GD fibroblasts. This result suggests that the nuclear translocation of endogenous IGF-1Rα may be limited by receptor fragmentation.

## Discussion

Several receptors initially thought to function at the cell surface have been subsequently found to traffic to the cell nucleus [36]. These include epidermal growth factor receptor (EGFR) [37], fibroblast growth factor receptor (FGFR) [38], [39], and Notch-1 [40]. EGFR can function in the nucleus as a transcription factor [37]. Cells and tissues with prominent nuclear EGFR localization exhibit accelerated proliferation. Moreover, EGF increases content of phosphorylated EGFR within the nucleus. Nuclear EGFR translocation following irradiation is tied to DNA repair and activation of DNA-dependent kinase [41]. A transactivation domain harbored by EGFR acts at specific target gene promoter sequences, such as the one identified on the cyclin D1 gene [37]. Like IGF-1R, EGFR exhibits intrinsic tyrosine kinase activity [42]. FGFR1 translocates to the nucleus following endocytosis provoked by FGF, through mechanisms linked to the activities of E-cadherin [39]. Balance between these two proteins on the surface of epithelial cells appears to be crucial to the nuclear accumulation of FGFR1. Once in the nucleus, FGFR1 interacts with the transcription co-activator, CREB-binding protein [43]. Unlike the actions of FGFR1 mediated at the plasma membrane, those occurring in nuclei of neural progenitor cells appear independent of endogenous tyrosine kinase activity. Rather, the FGFR1/CBP complex recruits RNA polymerase II and enhances histone acetylation [43]. From the findings reported here, it would appear that IGF-1R closely resembles Notch-1, another transmembrane protein that signals through intermediate proteins at the cell surface but can also traffic to the nucleus as a protease-generated cleavage fragment [40]. The cross linking studies here suggest that ^125^I IGF-1 nuclear IGF-1R also translocates from the cell surface (Fig. 5). It would appear that the nuclear IGF-1R is not synthesized *de novo*, since cycloheximide fails to block nuclear accumulation of the receptor provoked by IGF-1 or GD-IgG (data not shown). Previous studies by Vecchione *et al* [44] have demonstrated IGF-1Rβ trafficking to the proteosome where the protein can undergo ubiquination. This process is mediated through the Grb10 adapter protein which inhibits IGF-1R-dependent signaling [45] and serves to promote the interaction between IGF-1Rβ and Nedd4, a ubiquitin protein ligase, through the formation of a Grb10/DNedd4IGF-1Rβ complex [46]. Both proteosomal and lysosomal pathways are involved in IGF-1R degradation [44]. Thus while the turnover of the receptor has been characterized, the potential for its trafficking to the nucleus has not been reported previously. Examination of its primary structure reveals a single potential nuclear localization sequence ERKRRD located in the amino terminus of IGF-1Rβ. It remains uncertain whether that sequence is involved in IGF-1R trafficking. It is notable that IR can also be detected, albeit at low levels, in GD fibroblast nuclei (Fig. 2C). This finding is congruent with a recent report demonstrating that IR and IR substrate-1 associate with the nuclear matrix of insulin-treated osteoblast-like UMR-106 cells [47].

The current finding may have substantial clinical implications. Orbital fibroblasts from patients with GD exhibit a unique phenotype. Their distinctive characteristics may account for the susceptibility of the orbit to TAO. They express an unusual gene profile especially when treated with inflammatory cytokines [48]. They exhibit biosynthetic activities such as the exaggerated production of the glycosaminoglycan, hyaluronan [49], [50] and the exaggerated induction by cytokines of prostaglandin endoperoxide H synthase-2, the inflammatory cyclooxygenase [50]–[52]. TAO fibroblasts represent two distinct populations on the basis of their display of CD34 [53]. CD34^+^ fibroblasts appear to derive from circulating bone marrow fibrocytes which can differentiate into orbital fat cells. Recognizing that they over-express IGF-1R and exhibit divergent receptor trafficking and signaling could aid in developing targeted therapies.

**Figure 7:**
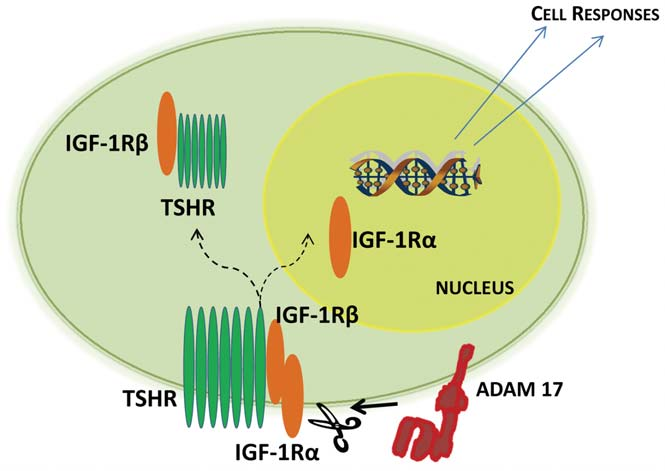
Schematic representation of the proposed translocation of IGF-1R. ADAM 17 acts to shed IGF-1R into fragments. IGF-1Rβ complexes with TSHR. IGF-1Rα translocates to the cell nucleus where it accumulates adjacent to chromatin. We hypothesize that it acts as a transcription factor, accounting for some of the cell responses peculiar to fibroblasts from patients with GD.

To the best of our knowledge, the current studies are the first to demonstrate IGF-1R fragmentation into differentially behaving proteins and nuclear translocation. Moreover, these findings implicate ADAM17 in the trafficking of this receptor. Fig. 7 contains the aggregate concept for how this non-canonical pathway for IGF-1R activity might explain the induction of IL-16 and RANTES by IGF-1 that occurs in a GD-specific pattern. The EGFR pathway also has been shown previously to be influenced by ADAM17 activity [28]. Specifically, the release and activation of multiple EGFR ligands is dependent on ADAM10 and ADAM17 [54]. These enzymes may play similar roles in IGF-1R activation and signaling. It is possible that the apparently high abundance of ADAM17 in GD orbital fibroblasts could explain, at least in part, the peculiar actions of IGF-1 and GD-IgG actions in these cells. Immediately following the initial submission of this paper, Sarfstein and colleagues reported that IGF-1R can translocate to the nucleus of breast cancer cells [55]. In so doing, the receptor can autoregulate *IGF-1R* gene expression by acting as a transcription factor. This potentially exciting set of findings may relate to the events outlined in the current communication.

Our current findings identify a heretofore unrecognized molecular signature displayed by GD orbital fibroblasts that appears to distinguish them from fibroblasts coming from healthy donors. Coupled with our recent observation that IGF-1Rβ forms a physical and functional complex with TSHR [25], the receptor can be implicated in complex processes associated with autoimmune orbital disease. Recognition of aberrant nuclear trafficking of IGF-1Rα in a disease-specific manner should allow examination of potentially important questions concerning its role in the pathogenesis of GD.

## Materials and Methods

### Materials

IGF-1R-blocking mAb 1H7 was obtained from BD Pharmingen (San Diego, CA). rhIGF-I was purchased from R&D Systems (Minneapolis, MN) or Calbiochem (San Diego, CA). Des (1–3) IGF-I was obtained from GroPep (Adelaide, Australia). TAPI-1 was from EMD Chemicals (Gibbstown, NJ). NVP-AEW541was generously provided by Novartis. Dexamethasone (1, 4 pregnadien-9-fluoro-16α-methyl-11β,17α, 21-triol-3,20-dione) and insulin were purchased from Sigma-Aldrich (St. Louis, MO). Polyclonal anti-IGF-1Rα and an anti-c-Jun Abs were from Santa Cruz Biotechnology (Santa Cruz, CA) while mAbs against IGF-1Rα and anti-Grb2 Abs were from US Biological, Swampscott, MA. Anti-IR mAb was supplied by Invitrogen (Camarillo, CA). Goat anti-rabbit secondary Ab conjugated with FITC and mounting medium containing propidium iodide (PI) or DAPI were obtained from VECTOR Laboratories (Burlingame, CA). Goat anti-rabbit secondary Ab conjugated with Oregon Green and goat anti-mouse secondary Ab conjugated with Texas-red were from Molecular Probes (Carlsbad, CA).

### IgG preparation

Sera were collected from patients with GD, either without or with clinical TAO, and from individuals without known thyroid disease (controls). Sera from a total of 3 patients with GD were used, and the diagnosis of GD was confirmed using clinical and laboratory-testing criteria as described previously [13]. These activities were approved by the Institutional Review Boards of the UCLA Center for Health Sciences, the Harbor-UCLA Medical Center, and the University of Michigan Health Care System. Written consent from all participants was obtained. IgG was prepared by protein A affinity chromatography [56].

### Cell culture

Human orbital fibroblasts were obtained from 6 individuals with GD and from 4 donors without known thyroid disease. Strains were initiated and propagated as described previously [57]. They were rigorously characterized and were free from contamination with smooth muscle, endothelial, and epithelial cells [58]. They exhibited a very stable phenotype and were utilized for experiments between the 2^nd^ and tenth passage from strain initiation.

For microscopy, fibroblasts were cultured on rat-tail collagen type 1 (BD Biosciences, Two Oak Park, MA)-treated cover glass in DMEM containing 10% FBS, antibiotics, and glutamine. Cells were passaged by gentle treatment with trypsin/EDTA in PBS and allowed to attach for 24 hours. They were then shifted to DMEM containing 1% FBS overnight. At least 2 h prior to other treatments, some cultures received dexamethasone (10 nM), IGF-1Rα-blocking mAb, 1H7 (5 µg/ml), NVP-AEW541 (0.5 µ M), or TAPI-1 (50 µM). They were then treated with nothing (control), IGF-1 (10 nM), control IgG (15 µg/ml) or GD-IgG (15 µg/ml) for the times indicated in the figure legends.

### Immunofluorescence and confocal imaging analysis

Immunofluorescence staining and confocal microscopy were performed as described previously [59]. Briefly, following the treatment periods specified, glass coverslips were fixed in 2% paraformaldehyde in PBS for 30 minutes and permeated with 0.2% Triton-X 100 for 30 minutes. These were then rinsed with PBS three times and incubated with the primary rabbit anti-IGF-1Rα mAb (1∶100) or anti-IGF-1Rβ (1∶100) Ab in PBST containing 5% goat serum for 2 hours. Subsequently, cover slips were rinsed with PBS and incubated with goat anti-rabbit secondary Ab conjugated with FITC (VECTOR) or Orgeon Green (Molecular Probe) for 45 minutes. Coverslips were rinsed extensively with PBS and mounted on glass slides with Vectashield mounting medium containing propidium iodide (VECTOR). Fibroblast images were acquired and analyzed using a Nikon Eclipse 800 microscope and a Nikon PCM 2000 two laser confocal system from Nikon Bioscience (Melville, NY). Some of the images were captured with a Zeiss Axioskop 40 microscope/AxioCamHRc camera. Average grey color intensity of nuclei was quantified randomly from at least 10 nuclei in each treatment group.

### Flow cytometry

Dissociated GD or control fibroblasts were bound by FITC-labeled anti-IGF-1Rα Ab for 30 minutes at 4°C and analyzed on a FACScan Flow Cytometer (BD BioSciences, San Diego, CA) as described in detail [23]–[25].

### Sub-cellular fractionation

Fibroblasts were shifted to DMEM without serum for 24 hours and then treated for 16 hours with IGF-1 (10 nM) or GD-IgG (15 µg/ml). Cellular fractionation was performed according to the protocol supplied with the NE-PER nuclear and cytoplasmic extraction reagents (Pierce, Rockford, IL). Fractions were boiled with SDS sample buffer, subjected to 7% SDS-PAGE, transferred to Immobilon-P membrane (Millipore, Billerica, MA) and probed as indicated with primary Abs followed by HRP-conjugated secondary Abs. Membranes were treated with ECL reagent (Amersham, Piscataway, NJ) and exposed to X-ray film (Pierce).

### Chemical cross-linking

Constant numbers of fibroblasts were seeded on to 100 mm in diameter tissue culture dishes and allowed to proliferate in DMEM without FBS for 24 hours. Plates were cooled and ice-cold DMEM containing ^125^I-IGF-1(1 µCi/ml) (ICN, Costa Mesa, CA)) was added for 30 minutes at 4°C. Medium was removed and either 2 mM Disuccinimidyl suberate (DSS) or 2 mM Bis(sulfosuccinimidyl suberate) (BS) (Pierce) in PBS was added to the dishes. Monolayers were incubated for 30 minutes at 4°C, washed, and DMEM without FBS was added for 16 hours. Cells were lysed, fractionated, and radioactivity measured with a Cobra II auto-gamma counter (Perkin-Elmer, Shelton, CT).

### Real-time PCR and construction of IGF-1Rα/GFP fusion adenovirus

Cellular RNA was collected, reverse transcribed and levels of ADAM17 mRNA were determined by real-time PCR as described previously [60]. The following primers: Forward 5′- GTTGGGTCTGTCCTGGTTTTC- 3′ and reverse 5′- GTCCATTCTCTGGTGGTCCAG- 3′.

An adenovirus containing the IGF-1Rα/GFP fusion protein, designated Ad-IGF-1Rα-GFP, was generated. Cellular RNA was purified from lysed orbital fibroblasts using RNA reagent from Biotecx (Houston, TX). RNAs were reverse-transcribed into cDNAs using an Omniscript RT kit (Qiagen, Valencia, CA). These cDNAs were utilized as templates for the PCR reaction to clone a fragment of IGF-1Rα. Primers used for the PCR were: forward 5′-GAATGAAGTCTGGCTCCGGAGG-3′ and reverse 5′-CTCTCCGCTTCCTTTCAGGTC-3′. The resulting 2.1 kb PCR fragment was TA cloned into pCR2.1 vector (Invitrogen, Carlsbad, CA) and further sub-cloned in-frame into a pEGFP-N1 vector (BD Biosciences, Franklin Lakes, NJ). This cDNA fragment was fused in-frame with GFP located positioned at the 3′ end, resulting in the generation of pEGFP-IGF-1Rα-GFP. To create an adenoviral expression vector containing IGF-1Rα and GFP, BD Adeno-X expression system 1 (Clontech) was utilized. IGF-1Rα–GFP fusion fragment was retrieved by cutting pEGFP-IGF-1R-GFP with NheI and ligated to the pshuttle2 vector. This plasmid was then cleaved with PI-SceI and I-CeuI. The IGF-1Rα–GFP fragment was ligated with Adeno-X viral vector, linearized with PacI, and used to transfect low-passage HEK 293 cells. CPE (Cytopathic effect) was evident 1 to 2 weeks after transfection. Cells with IGF-1Rα–GFP expression were visualized in green color. Viral particle-containing lysate was harvested by freeze-thawing cells and stock was used to infect fibroblasts with an m.o.i. of at least 100.
